# Machine Learning Approach Identified Multi-Platform Factors for Caries Prediction in Child-Mother Dyads

**DOI:** 10.3389/fcimb.2021.727630

**Published:** 2021-08-19

**Authors:** Tong Tong Wu, Jin Xiao, Michael B. Sohn, Kevin A. Fiscella, Christie Gilbert, Alex Grier, Ann L. Gill, Steve R. Gill

**Affiliations:** ^1^Department of Biostatistics and Computational Biology, University of Rochester Medical Center, Rochester, NY, United States; ^2^Eastman Institute for Oral Health, University of Rochester Medical Center, Rochester, NY, United States; ^3^Department of Family Medicine, University of Rochester Medical Center, Rochester, NY, United States; ^4^Microbiology and Immunology, University of Rochester Medical Center, Rochester, NY, United States

**Keywords:** machine learning, statistical approaches, dental caries, multiplatform analysis, candida, oral microbiome

## Abstract

Untreated tooth decays affect nearly one third of the world and is the most prevalent disease burden among children. The disease progression of tooth decay is multifactorial and involves a prolonged decrease in pH, resulting in the demineralization of tooth surfaces. Bacterial species that are capable of fermenting carbohydrates contribute to the demineralization process by the production of organic acids. The combined use of machine learning and 16s rRNA sequencing offers the potential to predict tooth decay by identifying the bacterial community that is present in an individual’s oral cavity. A few recent studies have demonstrated machine learning predictive modeling using 16s rRNA sequencing of oral samples, but they lack consideration of the multifactorial nature of tooth decay, as well as the role of fungal species within their models. Here, the oral microbiome of mother–child dyads (both healthy and caries-active) was used in combination with demographic–environmental factors and relevant fungal information to create a multifactorial machine learning model based on the LASSO-penalized logistic regression. For the children, not only were several bacterial species found to be caries-associated (*Prevotella histicola, Streptococcus mutans*, and *Rothia muciloginosa*) but also *Candida* detection and lower toothbrushing frequency were also caries-associated. Mothers enrolled in this study had a higher detection of *S. mutans* and *Candida* and a higher plaque index. This proof-of-concept study demonstrates the significant impact machine learning could have in prevention and diagnostic advancements for tooth decay, as well as the importance of considering fungal and demographic–environmental factors.

## Introduction

Poor maternal and child oral health is a public health crisis with potential intergenerational health impacts. With oral disease affecting 50% of the global population (3.9 billion) and untreated tooth decay (dental caries) impacting almost half of the world’s population (44%), oral disease has become the most prevalent of all the 291 conditions included in the Global Burden of Disease Study (FDI World Dental Federation. https://www.fdiworlddental.org/oral-health/ask-the-dentist/facts-figures-and-stats. Accessed September 5, 2020). Significantly, children younger than 5 years and their mothers, who together comprise 22% of the whole population, are profoundly affected by dental caries. Unmet oral health needs worsen among minority women and children who are from low-income families (Marchi et al., 2010; Thompson et al., 2013; Singhal et al., 2014).

Dental caries is multifactorial infectious disease, initiated from the virulent dental biofilms/plaque formed on tooth surfaces (Tanzer, 1995). Within dental biofilms/plaque, oral cariogenic bacteria metabolize dietary carbohydrates resulting in acid production and initiating demineralization of tooth enamel (Bowen, 2016). Remineralization, or restoration of mineral ions, is mediated through salivary calcium, phosphate, and fluoride ions. In a healthy (caries-free) mouth, the remineralization and demineralization rates are at equilibrium; when the demineralization rate exceeds the remineralization rate, tooth decay occurs (Takahashi and Nyvad, 2011; Abou Neel et al., 2016). Often, this shift from equilibrium is caused by a disruption in the ecology of the oral microbiome from a largely commensal community to a community dominated by cariogenic bacteria.

Recognizing the essential contribution of oral microorganisms to dental caries, the development of effective predictive models that utilize sensitive microbial markers would offer substantial opportunities to predict and prevent caries. However, because of the multifactorial etiology of dental caries, developing effective predictive models is also challenging. The current dental caries prediction model falls into two categories: 1) one utilizing classical statistical models that assess the contribution of demographic and environmental factors, either without consideration of microbial factors or only including a limited number of traditional caries risk markers, e.g., *Streptococcus mutans* and *Lactobacillus* (Caufield et al., 1993; Klein et al., 2004; Li et al., 2005; Kanasi et al., 2010; Slayton, 2011; Zhan et al., 2012; Klinke et al., 2014); 2) the other utilizing statistical/machine learning models that identify caries-related taxa and its differential abundance based on caries status, with limited adjusting of demographic, environmental, and other contributing factors (Teng et al., 2015; Grier et al., 2020). The approaches mentioned above do not take caries multifactorial etiology into account. Furthermore, in the past decade, studies have also indicated the potential cariogenic role of *Candida albicans* in children, together with *S. mutans* (Hossain et al., 2003; de Carvalho et al., 2006; Rozkiewicz et al., 2006; Raja et al., 2010; Srivastava et al., 2012; Yang et al., 2012; Klinke et al., 2014; Qiu et al., 2015; Alkhars et al., 2021). However, the existing caries prediction models have not assessed the contribution of *Candida*. Therefore, developing a statistical/machine learning model that assesses all caries-related risk factors, including bacteria, *Candida*, and demographic-environmental factors, is urgently needed.

To address this research gap, as a proof-of-concept study, we developed statistical/machine learning (ML) models to identify caries-related oral microbes in cross-sectional mother-child dyads from a low-income underserved background.

## Materials and Methods

### Study Population, Sample Collection, and 16S Ribosomal RNA Sequencing Data

A cohort of mother-child dyads with a balanced distribution of children with or without early childhood caries (ECC) was enrolled at the Eastman Institute for Oral Health, University of Rochester, detailed previously (Xiao et al., 2018a). Ethical approval of the study was obtained from the University of Rochester Research Subject Review Board (RSRB00056870). Children were younger than 6 years. Subjects who had severe systematic diseases or antibiotic treatment within the previous 3 months were excluded. Non-stimulated whole saliva was collected from subjects through a saliva jet connected to a suction pump at least 2 h after any tooth brushing, eating, or drinking. Supragingival dental plaque was collected from the whole dentition with a standard dental scaler. Previous established methods were used to isolate and identify *Candida species* (Xiao et al., 2016), and to perform oral microbiome sequencing and related bioinformatics analysis (Merkley et al., 2015; Grier et al., 2017). The results of the 16S ribosomal RNA (16s rRNA) sequencing data were detailed previously (Xiao et al., 2018a). Sequencing data that passed quality controls were included in this study to develop caries predictive model and were assigned to operational taxonomic units (OTUs) using the 2014 release of the closed reference OSU CORE database (Griffen et al., 2011). DESeq2-negative binomial Wald test was used to compare the microbial differential abundance at species level between caries and caries-free groups among the children and their mothers.

### Variables

The primary outcome is caries status (Y/N). The independent variables were as follows: (1) race (Black/African American or other), (2) years of age (ordinal), (3) ethnicity (Hispanic or non-Hispanic), (4) tooth brushing frequency (0, not every day; 1, once per day; 2, two times per day), (5) daycare attendance (Y/N), (6) inhaler use (for children only), (7) Plaque index (ordinal), (8) oral *Candida* status (Y/N), (9) relative abundance of taxa. Demographic-socioeconomic and oral hygiene behavior characteristics were collected through questionnaires.

### Transformation of Relative Abundance

The centered log-ratio (CLR) transformation was applied to the relative abundance of taxa, where for each subject, the sample vector undergoes a transformation based on the logarithm of the ratio between the individual elements and the geometric mean of the vector. CLR removes the value-range restriction of percentages (relative abundance is a percentage) but keeps the sum constraint of compositional data.

### LASSO-Penalized Logistic Regression

Our goal is to build a model that could explain and predict the probability of having caries based on a small set of factors. Therefore, logistic regression model was fitted for the response variable whether the subject has caries or not, on a large pool of candidate input variables, including demographic and clinical factors and CLR-transformed relative abundance of taxa. Because the number of candidate variables (~360) far exceeds the number of subjects (~40 in each model), regularization is needed to avoid overfitting and to identify a small set of relevant variables. Variable selection technique, specifically LASSO penalty, was applied. K-fold cross-validation (K=4) was used to determine the optimal value of the tuning parameters for the LASSO penalty, i.e., the strength of the selection. Using this tuning parameter, the model was fitted and the solution path was calculated to show the order of variables entering the model.

## Results

### Caries Prediction Models for Children

**Figure 1:**
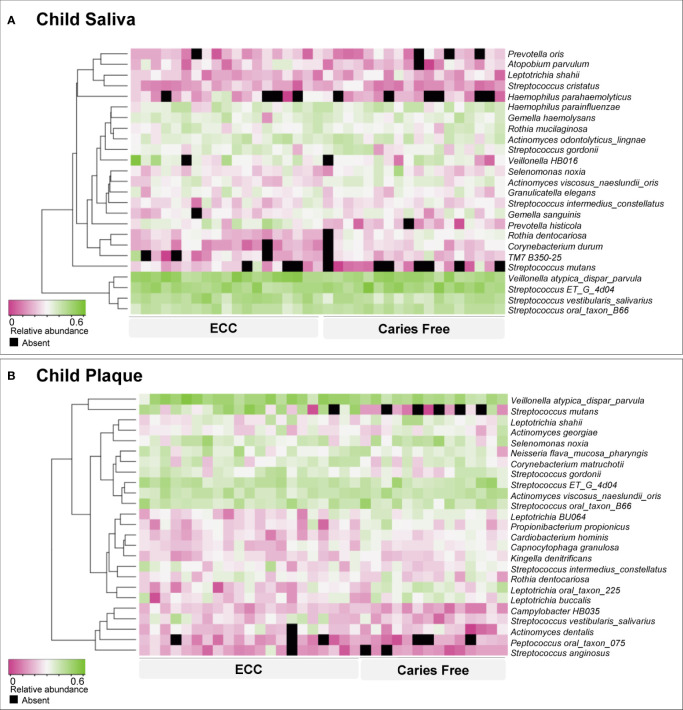
Early childhood caries-associated and caries-free–associated oral microbiome in children. Based on relative abundance, the salivary **(A)** and plaque **(B)** microorganisms were clustered into ECC-associated and caries-free–associated groups, as shown by the dendrogram on the left. Relative abundance is indicated by a gradient of shades from pink to green. Black spots indicate no detection of the species.

For the children’s model, species-level sequencing data of 37 salivary samples and 36 plaque samples were used. The salivary and plaque microbial profiling is shown in **Figure 1**. *Veillonella atypica_dispar_parvula* and *Streptococcus ET_G_4D04* are the most abundant species in children’s saliva and plaque. Not surprisingly, *S. mutans* is more abundant and with a higher detection among children with ECC.

#### Saliva Model

**Figure 2:**
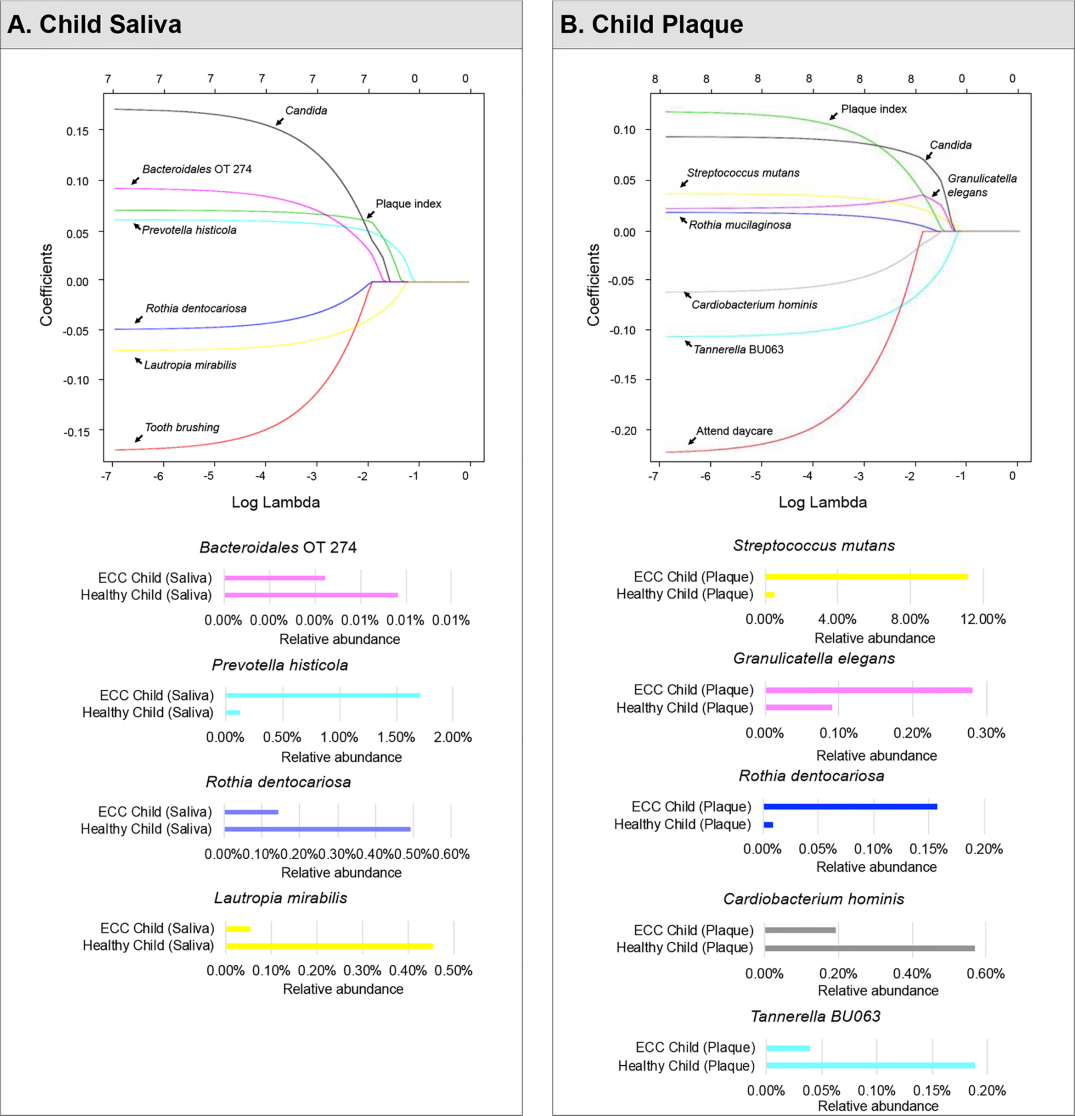
Identified factors associated with child’s caries risk using machine Learning model. LASSO-penalized logistic regression modeling was used for caries predictor selection for children’s saliva and plaque samples. Specifically, seven variables for models using salivary microorganisms **(A)** and eight variables for models using plaque microorganisms **(B)** were identified as predictive factors for dental caries in preschool children. The LASSO solution path above shows how the model is built sequentially by adding one variable at a time to the active set.

Using 37 children’s saliva taxa data (relative species abundance), we ran a LASSO-penalized logistic regression model on having caries (1) or not (0) on a pool of candidate variables including 353 species in the microbiome data, four demographic variables (age, gender, race, ethnicity), four medical–dental–behavior characteristics (frequency of toothbrushing per day, attending daycare, inhaler use, and plaque index), and one fungal-related parameter (*Candida* detection status). Seven variables were identified to be associated with caries. The LASSO solution path (**Figure 2A**) shows how the model is built sequentially by adding one variable at a time to the active set (i.e., set of variables with non-zero coefficients).

In a sequential order, *Prevotella histicola* (1.7% in ECC children and 0.12% in caries-free children) was the first variable that enters the saliva model, indicating that if a model with only one variable is desired, *P. histcola* would be the one to be used. As lambda decreases, *Lautropia mirabilis* entered the model as the second variable. Then plaques index and *Candida* were selected into the model, followed by an unclassified *Bacteroidales* oral taxon 274, *Rothia dentocariosa*, and toothbrushing frequency.

Moreover, *P. histicola*, plaque index, *Candida*, and *Bacteriodales* Oral Taxon 274 were predicted to be associated with an increased risk for caries; whereas, *L. mirabilis*, toothbrushing, and *R. dentocariosa* were associated with a decreased risk for caries in children. When lambda reached an extremely small number (e.g., 10^-7^ in the far left of the solution path), the coefficient estimates are approximately the same as those in the unpenalized logistic regression model. The predictive model using childrens’ saliva samples is given by:

$$\begin{array}{c} logit(p)=X\beta =0.641+0.174Candida-0.170toothbrush+0.072plaque\\ -0.048Rothia\;dentocariosa+0.062Prevotella\;histicola\\ +0.094Bacteroidales\;oral\;taxon\;274-0.069Lautropia\;mirabilis, \end{array}$$

where the probability of having caries can be estimated by

$$p(caries)=\frac{\exp(X\beta )}{1+\exp(X\beta )}$$

Using the variable “toothbrush” as an example, the interpretation of the coefficient is that for individuals who brush their teeth for one or more times per day, the odds of having caries will be exp(−0.170)=0.84 times the odds for those who do not, which will result in an approximately 16% reduction. Similarly, individuals who brush their teeth twice per day will have an odds of 30% lower than those who do not brush teeth every day.

**Figure 3:**
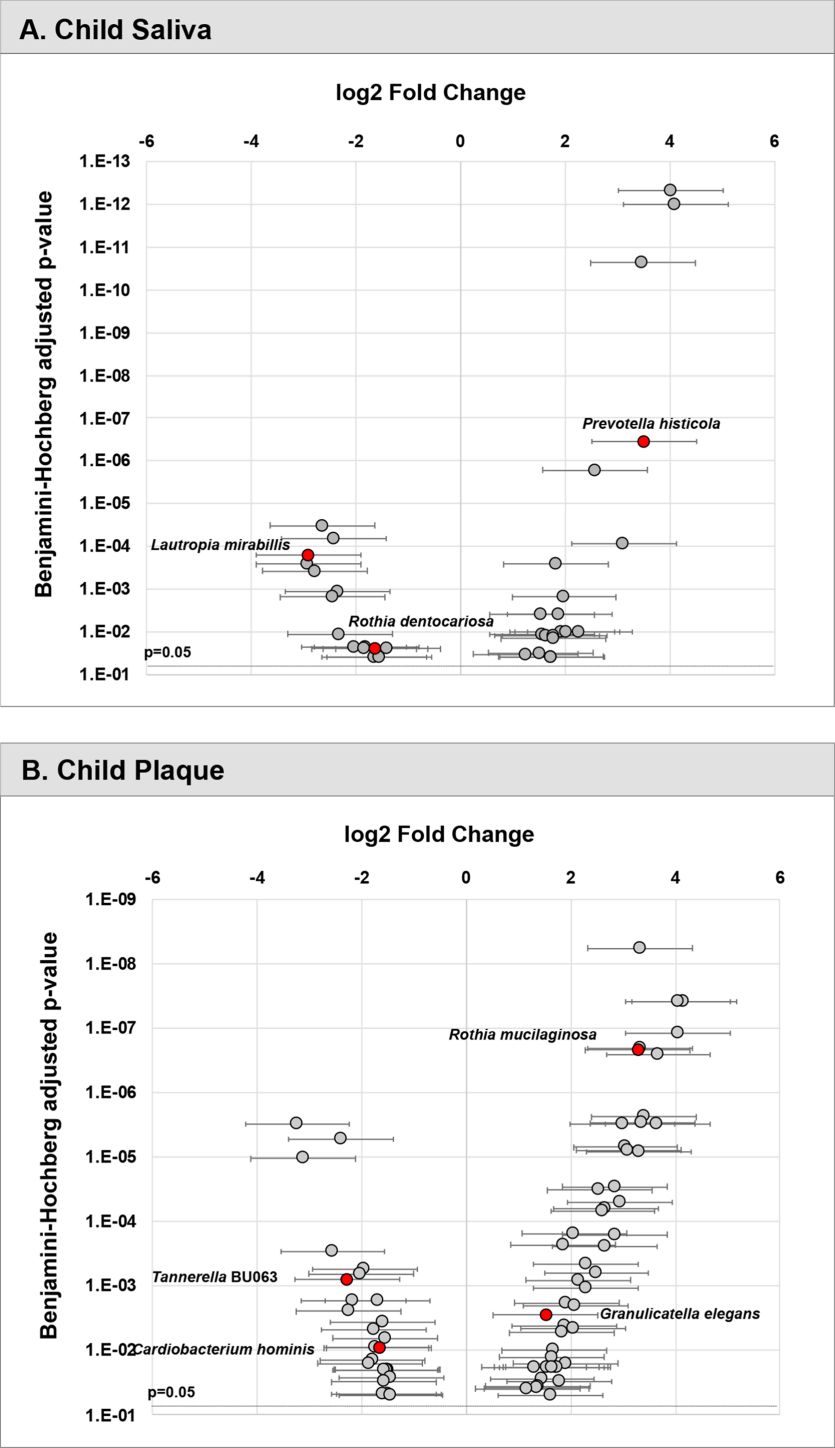
Differential abundance of taxa in children’s saliva and plaque. Relative fold change in abundance of species in saliva **(A)** and plaque **(B)** from children with ECC *vs.* caries-free. All species plotted with a p value <0.05.

Furthermore, the differential abundance of three selected species (*L. mirabillis*, *R. dentocariosa*, *P. histicola*) was statistically significant (p<0.05) (see **Figure 3A**).

#### Plaque Model

In the plaque model (**Figure 2B**), eight variables were selected, with five of them being the relative abundance of bacterial taxa, one fungal-related (*Candida* status), one oral hygiene index, and one behavior parameter. In sequential order, *S. mutans* entered the model as the first variable with an increased risk for caries (11.14% in ECC and 0.47% in caries-free children). *Tannerella BU063* entered the model as the second variable with a reduced caries risk, more abundant in caries-free children (0.18%), and less abundant in ECC children (0.04%). *Candida* (increased caries risk) was the third variable selected, followed by *Granulicatella elegans* (increased caries risk), plaque index, *Cardiobacterium hominis* (reduced caries risk), *Rothia mucilaginosa* (increased caries risk), and daycare attendance (reduced caries risk). The differential abundance of four selected species (*T.* BU063, *R. mucilaginosa*, *C. hominis, and G. elegans*) was statistically significant (p<0.05) (see **Figure 3B**).

The predictive model that used children’s **plaque** samples is the follows:

$$\begin{array}{c} logit(p)=X\beta \\ =0.459+0.098Candida-0.231daycare\;attendance+0.125plaque\\ +0.020Rothia\;mucilaginosa-0.110Tanneralla\;BU063\\ +0.024Granulicatella\;elegans+0.039Streptococcus\;mutans\\ -0.063Cardiobacterium\;hominis \end{array}$$

### Caries Prediction Model for Mothers

**Figure 4:**
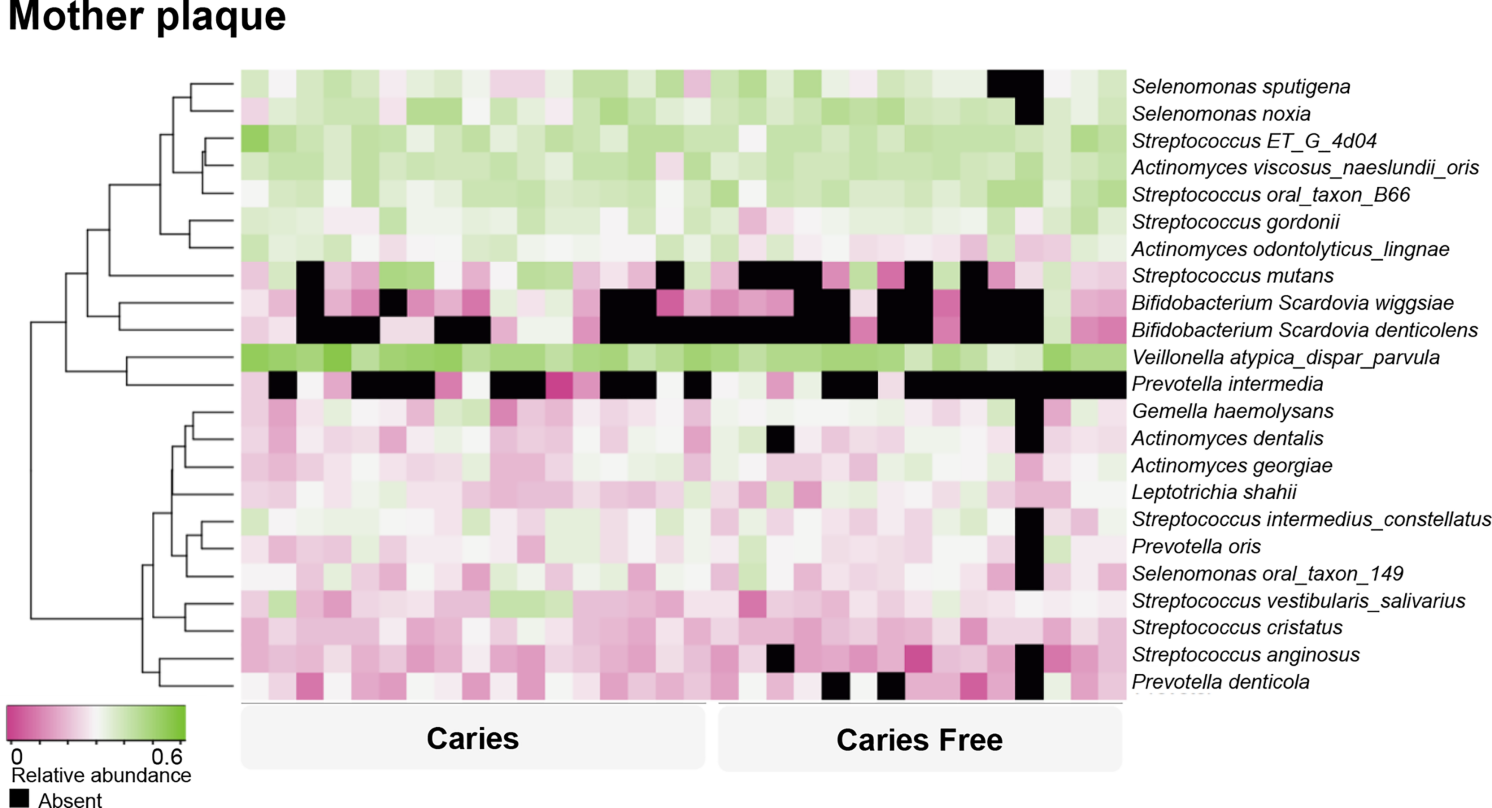
Caries-associated and health-associated plaque microbiome in mothers. Based on their relative abundance, the supragingival plaque microorganisms were clustered into caries associated and caries-free–associated groups, as shown by the dendrogram on the left. Relative abundance is indicated by a gradient of shades from pink to green. Black spots indicate no detection of the species.

**Figure 5:**
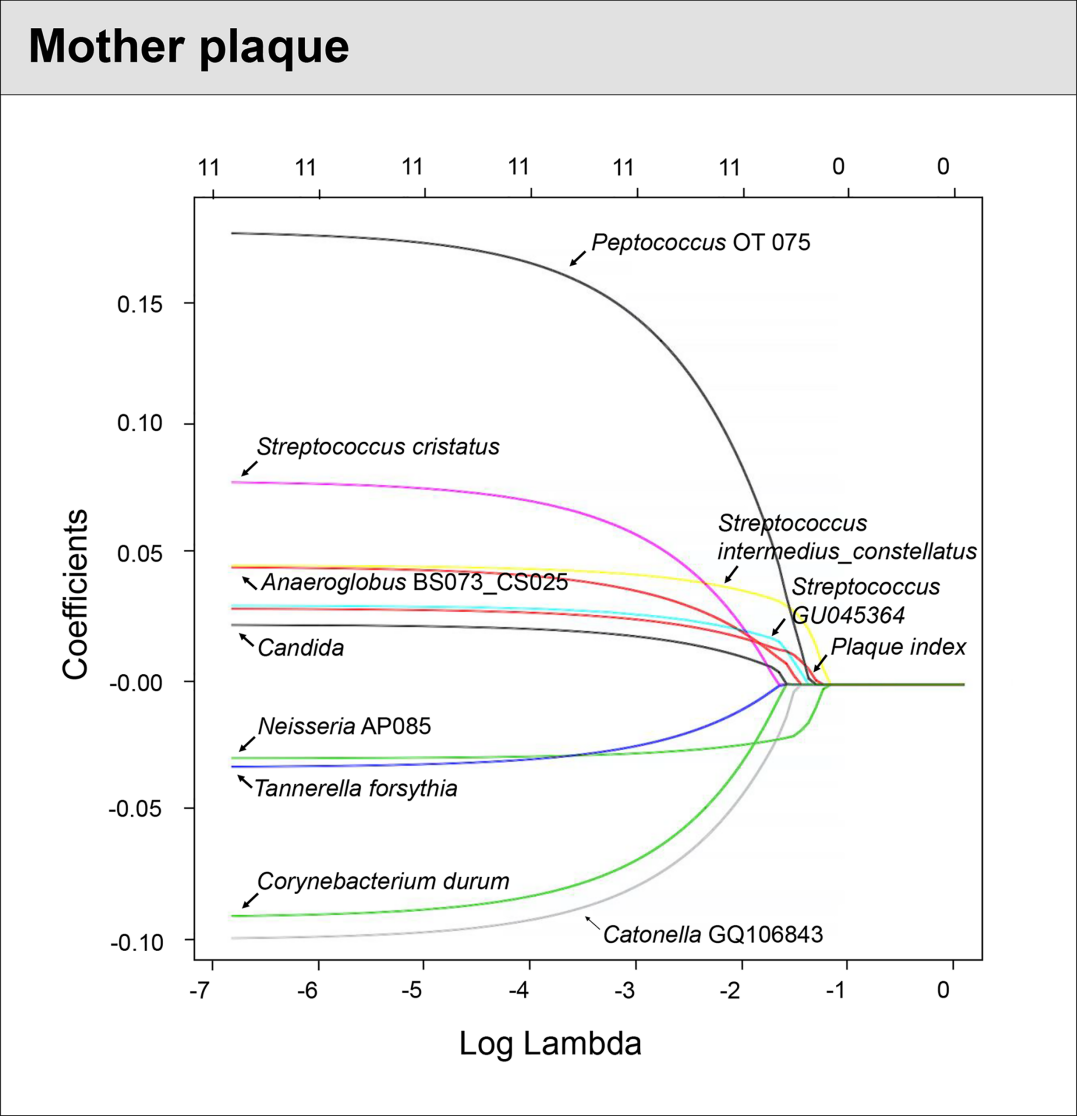
Identified factors associated with mother’s caries risk using machine Learning model. LASSO-penalized logistic regression modeling was used for caries predictor selection for children’s saliva and plaque samples. Eleven variables for models using plaque microorganisms were identified as predictive factors for dental caries in mothers. The LASSO solution path above shows how the model is built sequentially by adding one variable at a time to the active set.

For the mothers’ model, species-level sequencing data of 32 plaque samples were used. The plaque microbial profiling of each sample is shown in **Figure 4**. For the mothers, the LASSO solution path that demonstrates how the model is built sequentially is shown in **Figure 5**. Nine taxa, one fungal parameter (*Candida* status) and oral hygiene index were selected into the model. *Streptococcus intermedius_constellatus* (increased caries risk) and *Neisseria* AP085 (decreased caries risk) entered the model closely as the top 2 variables. Plaque index (increased risk) was selected as the third variable in the model. The remaining variables were selected in an order as follows: *Peptococcus* OT 075 (increased risk), *Streptococcus* GU045364 (increased risk), *Anaeroglobus* BS073_CS025 (increased risk), *Catonella* GQ106843 (decreased risk), *Candida* (increased risk), *Corynebacterium durum* (decreased risk), *Streptococcus cristatus* (increased risk), and *Tanneralla forsythia* (decreased risk).

The predictive model that used mothers’ **plaque** samples is the following:

$$\begin{array}{c} logit(p)=X\beta \\ =0.459+0.006Candida+0.012plaque\\ -0.022Corynebacterium\;durum-0.002Tannerel\;forsythia\\ +0.018Streptococcus\;GU045364+0.003Streptococcus\;cristatus\\ +0.033Streptococcus\;intermedius\_constellatus\\ -0.023Catonella\;GQ106843+0.052Peptococcus\;oral\;taxon\;075\\ +0.014Anaeroglobus\;BS073\_CS025-0.009Neisseria\;AP085 \end{array}$$

### Performance of Caries Prediction Model

**Figure 6:**
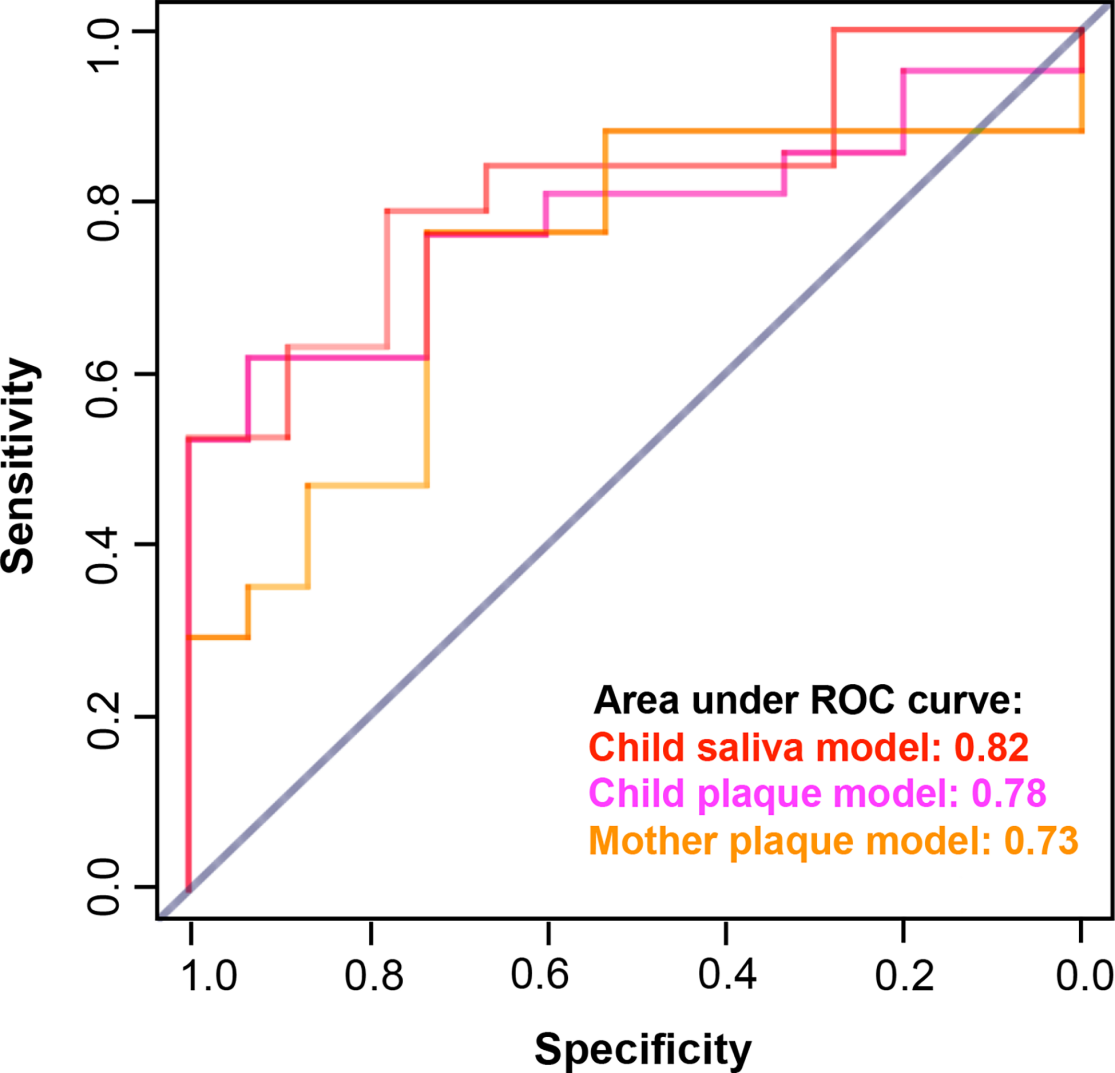
Performance of caries prediction models. The caries prediction models achieved desirable performance that was assessed by area under the ROC curve (AUC). The AUC for the child saliva model was 0.82; The AUC for the child plaque model was 0.78; and the AUC for the mother plaque model was 0.73.

The caries prediction models achieved desirable performance that was assessed by area under the ROC curve (AUC), see **Figure 6**. The average AUC over 20 random four-fold cross-validation was 0.82 for the child’s saliva model, 0.78 for the child’s plaque model, and 0.73 for the mother’s plaque model.

## Discussion

ML modeling of the oral microbiome has the potential to identify microbial biomarkers and aid in the prediction of dental caries. This proof-of-concept study is the first study to our knowledge that used an enhanced ML approach to incorporate multi-source variables with microbial data for dental caries prediction. The developed models have the following features:

1. The models considered the multifactorial etiology of dental caries by incorporating demographic-medical-dental characteristics, oral hygiene practice, daycare attendance, together with 16s relative species abundance, and oral *Candida* status. A meta-analysis indicates that children with oral *C. albicans* have (>5 times) higher odds of having ECC compared with those without *C. albicans* (Xiao et al., 2018b) and children with early-life oral *Candida* colonization are at higher risk for *S. mutans* emergence in the mouth by 1 year of age (Alkhars et al., 2021). However, no caries risk models have reflected the potential contribution from *Candida*. Our study demonstrated the consistent contribution from *Candida* in both child’s and mother’s prediction model.
2. The sequential order of the variables that entered the model reflects the contribution of the variables to predicting dental caries.
3. Intercept of the variables in the model would enable quantification of caries risk assessment, which has important clinical use implication.

Teng et al. (2015) used temporal patterns of the salivary and plaque microbiome to predict onset of ECC among children followed up from 4 to 6 years of age. The most discriminate species in Teng’s predictive model included *S. mutans* and *P. histicola*. Our model also included *P. histicola* as a discriminate species with an increased abundance in caries-active children, this is supported by previous findings of the association of *P. histicola* and caries (Hurley et al., 2019). Although *P. histicola* has been identified in several studies as being associated with caries and lower pH values, its direct function and role in the oral microbiome has not been elucidated. Additionally, Grier et al. (2020) developed a ML model to identify species in saliva that is associated with the onset of ECC among 56 preschool children. *R. mucilaginosa*, *Streptococcus* sp. and *Veillonella parvula* were selected as discriminatory markers for ECC onset. Both *Streptococcus* sp. and *Veillonella parvula* were highly abundant in the plaque and saliva samples of children with caries in our study. The early colonizer *R. mucilaginosa* was not a discriminatory microbe in our salivary model; this may be reflective of the age of our cohort and demonstrates a need for studies at earlier timepoints (and longitudinally).

Despite having a smaller sample size in our model, the performance of our ML was comparable or better than models of these studies. Potentially, this could be because of the advantages of our multifactorial approach. This is exemplified by *Candida* presence (increased risk) and toothbrushing frequency (decreased risk) as strong discriminatory factors in our model, which would have been otherwise missed if our ML relied solely on 16s rRNA data. In addition, our model was able to utilize both cariogenic and protective bacteria as discriminatory markers. *Lautropia mirabilis* and *Rothia dentocariosa* were found to be protective markers in the salivary microbiome of the children in this study. In a comparison of caries-free and caries-active children of 6 to 9 years old, *L. mirabilis* was found to have a significantly higher abundance in the caries-free children (Qudeimat et al., 2021). Future *in vitro* assays would be helpful to determine whether *L. mirabilis* functions to create a healthier (neutral pH) oral environment, or if a higher abundance of *L. mirabilis* implies an indirect consequence of an already neutral pH. Additionally, a higher abundance of *R. dentocariosa* has been found in children with caries in some studies (Jiang et al., 2016; Inquimbert et al., 2019), which is contrary to our results.

Using the proposed ML models, we identified specific caries-related oral bacteria, *Candida*, together with other multi-source factors for preschool children and their mothers. Prediction models for both children and their mothers achieved desirable performance. Fine-tuning and further validation are needed using larger and longitudinal caries onset sample set. Future models will consider incorporating more diverse biological and environmental variables, including children’s diet, parent’s education, oral hygiene, health, and oral microbiome. Experimental and clinical confirmation of the predicted microbial signatures for caries risk prediction and the translation into clinically measurable parameters of antigen and bacterial abundance will enhance our ability to identify at-risk children and promote the development of preventative therapeutics.

The following limitations need to be considered when interpreting the study results: (1) limited sample size; (2) conducted in one US city. Thus, generalization to other populations is unreliable because of the small convenient sample size; (3) with the data set being cross-sectional data set, the models are built upon the existing caries status, not through the longitudinal onset of caries. Future validations of our models are warranted using longitudinal data set.

## Data Availability Statement

The original contributions presented in the study are included in the article material. Further inquiries can be directed to the corresponding authors.

## Ethics Statement

The studies involving human participants were reviewed and approved by University of Rochester Research Subject Review Board. Written informed consent to participate in this study was provided by the participants or the participants’ legal guardian.

## Author Contributions

TTW, JX, MBS, KAF, CG, and SRG contributed to the conception, design, data acquisition, analysis, and interpretation, drafting, and critically revising the manuscript. AG and ALG contributed to data acquisition, analysis, and critically revising the manuscript. All authors contributed to the article and approved the submitted version.

## Funding

JX’s research was supported by the National Institute of Dental and Craniofacial Research grant K23DE027412. TT’s work is supported by grant from the National Science Foundation NSF-CCF-1934962. 

## Conflict of Interest

The authors declare that the research was conducted in the absence of any commercial or financial relationships that could be construed as a potential conflict of interest.

## Publisher’s Note

All claims expressed in this article are solely those of the authors and do not necessarily represent those of their affiliated organizations, or those of the publisher, the editors and the reviewers. Any product that may be evaluated in this article, or claim that may be made by its manufacturer, is not guaranteed or endorsed by the publisher.
